# The expectations humans have of a pleasurable sensation asymmetrically shape neuronal responses and subjective experiences to hot sauce

**DOI:** 10.1371/journal.pbio.3002818

**Published:** 2024-10-08

**Authors:** Yi Luo, Terry Lohrenz, Ellen A. Lumpkin, P. Read Montague, Kenneth T. Kishida

**Affiliations:** 1 Shanghai Key Laboratory of Mental Health and Psychological Crisis Intervention, School of Psychology and Cognitive Science, East China Normal University, Shanghai, China; 2 Fralin Biomedical Research Institute, Virginia Tech, Roanoke, Virginia, United States of America; 3 NYU-ECNU Institute of Brain and Cognitive Science at NYU Shanghai, Shanghai, China; 4 Department of Cell and Molecular Biology, Helen Wills Neuroscience Institute, UC Berkeley, Berkeley, California, United States of America; 5 Department of Physics, Virginia Tech, Blacksburg, Virginia, United States of America; 6 Department of Translational Neuroscience, Wake Forest University School of Medicine, Winston-Salem, North Carolina, United States of America; 7 Department of Neurosurgery, Wake Forest University School of Medicine, Winston-Salem, North Carolina, United States of America; Institute of Psychology Chinese Academy of Sciences, CHINA

## Abstract

Expectations shape our perception, profoundly influencing how we interpret the world. Positive expectations about sensory stimuli can alleviate distress and reduce pain (e.g., placebo effect), while negative expectations may heighten anxiety and exacerbate pain (e.g., nocebo effect). To investigate the impact of the (an)hedonic aspect of expectations on subjective experiences, we measured neurobehavioral responses to the taste of hot sauce among participants with heterogeneous taste preferences. By identifying participants who “liked” versus those who strongly “disliked” spicy flavors and by providing contextual cues about the spiciness of the sauce to be tasted, we dissociated the effects of positive and negative expectations from sensory stimuli (i.e., visual and gustatory stimuli), which were the same across all participants. Our results indicate that positive expectations lead to modulations in the intensity of subjective experience. These modulations were accompanied by increased activity in brain regions previously linked to information integration and the placebo effect, including the anterior insula, dorsolateral prefrontal cortex, and dorsal anterior cingulate cortex, as well as a predefined “pleasure signature.” In contrast, negative expectations decreased hedonic experience and increased neural activity in the previously validated “Neurological Pain Signature” network. These findings demonstrate that hedonic aspects of one’s expectations asymmetrically shape how the brain processes sensory input and associated behavioral reports of one’s subjective experiences of intensity, pleasure, and pain. Our results suggest a dissociable impact of hedonic information: positive expectations facilitate higher-level information integration and reward processing, while negative expectations prime lower-level nociceptive and affective processes. This study demonstrates the powerful role of hedonic expectations in shaping subjective reality and suggests potential avenues for consumer and therapeutic interventions targeting expectation-driven neural processes.

## Abstract 2

Can positive and negative expectations of a sensory experience cause neuronal and perception differences? This study shows that sensory experiences and neuronal responses are shaped by whether a person likes spicy foods, suggesting that perception could be pharmacologically modulated.

## Introduction

Expectations, triggered by contextual signals in the environment, have a profound impact on how humans make sense of and react to the world [1]. Expectations refer to beliefs about events that are likely to be experienced in the near future and may be associated with positive or negative valence. On the one hand, expecting a positive outcome can reduce emotional distress and relieve pain [2,3]. On the other hand, expecting a negative outcome can induce anxiety and enhance the experience of pain [4]. Such effects occur commonly in clinical settings—positive expectations can produce beneficial outcomes of treatments (i.e., placebo effect), whereas negative expectations can lead to poorer outcomes (i.e., nocebo effect) [5]. Further, the effects of (an)hedonic expectations also be observed in our daily experiences, including how we experience the foods eat and the events we experience in our social networks [6].

Placebo analgesia and nocebo hyperalgesia are examples of how positive or negative expectations can relieve or enhance, respectively, human sensory experiences. Although both placebo and nocebo effects exert modulations in the insula and dorsal anterior cingulate cortex (ACC) [7], they engage distinct brain networks [8]. The placebo effect was found to engage the dorsolateral prefrontal cortex (DLPFC) and the reward-based substrates [9–11], whereas the nocebo effect was associated with increased activity of the hypothalamic–pituitary–adrenal axis [5].

Recent placebo and nocebo studies have demonstrated how positive and negative expectations can modulate sensory and emotional responses and highlight the complexity of expectation effects and the interplay between different neural circuits, including endogenous opioid and descending pain modulatory system (DPMS), as well as reward system [12–14]. For example, opioid-rich descending pain modulatory structures such as the rostral ACC, amygdala, and periaqueductal gray play a key role in both placebo and nocebo effects, mediating modulation of pain through cognitive and emotional pathways [15]. Additionally, the reward circuit, including structures such as the nucleus accumbens and the ventral tegmental area, has been implicated in the positive effects of placebo expectations, further underscoring the role of motivation and reward in pain perception [16].

A recent meta-analysis found only small placebo effects on a neurologic pain signature (NPS), a neural measure that sensitively and reliably tracks changes in nociceptive input and self-reports of pain that arise from it [17]. As the NPS (which includes lateral somatosensory and medial limbic regions) is more closely tied to primary nociceptive/affective aspects of cognitive pain modulation [18,19], it seems plausible that modulation by positive expectations may involve higher-level cognitive and affective processes rather than direct primary nociceptive processing. Further, regions associated with negative affect and pain processing (e.g., dorsal ACC and posterior insula) have been found involved in nocebo manipulations compared to placebo ones [14]. The overlap between these regions and the NPS suggests that negative expectations exert their effect in earlier nociceptive/affective processes than do positive expectations; however, this hypothesis has yet to be tested by empirical studies.

One’s expectations can be complex compositions that include both sensory and hedonic components. Sensory expectations adhere to stimulus qualities, such as sensory modality and intensity. By contrast, hedonic expectations are beliefs about the degree to which one will like/dislike a stimulus [20]. A given sensory cue, even while generating identical sensory expectations, can form different hedonic expectations (i.e., positive or negative valence) in different individuals or contexts. Take the case of “spicy foods” as an example, while participants may anticipate the same external sensory input in terms of the physical properties of the spicy sauce, such as its visual, somatosensory, and olfactory features, the emphasis placed on these sensory cues may vary according to individual preferences, potentially shaping their hedonic expectations: someone who enjoys spicy food will expect a favorable experience associated with positive emotions, while someone who dislikes spicy foods will likely have an aversive expectation associated with negative emotions. In previous studies comparing positive and negative expectations, commonly used stimuli also differ in sensory intensity expectations, for example, in investigations regarding pain relief versus pain exacerbation [7,11]. It remains unknown how positive and negative expectations resulting from the same sensory experience might engage different neural circuits, leading to individual differences in people’s subjective intensity and hedonic experiences.

To address the question of how subjective experiences and neural responses are modulated by positive versus negative expectations (with expectations of sensory intensity controlled), we used a behavioral paradigm where participants continuously rated their subjective experience of stimulus intensity and variations in their associated hedonic experience while ingesting squirts of high- and low-intensity hot sauces or water. As an objective sensory input, we used hot sauces with variable, but quantifiable, amounts of capsaicin, the pungent ingredient in chili peppers that activates TRPV1 receptors on nociceptive neurons to produce a sensation of burning. This input allowed us to selectively activate a robust, receptor-specific sensory experience while controlling the intensity of an objective sensory cue [21]. Unlike earlier studies, which often focused on a single aspect of pain perception, our design allows for a simultaneous assessment of both sensory intensity and preference ratings. This dual assessment provides a more comprehensive picture of how expectations influence pain experiences. More importantly, by determining individual differences in preference for spicy flavor, we tested whether and how positive versus negative expectations about the same sensory input differentially modulate people’s sensory and hedonic experiences. Using functional magnetic resonance imaging (fMRI), we also provide evidence about how neural representations are altered by expectations. We hypothesized that even when the expected stimuli are identical, positive expectations predominantly modulate sensory intensity experience and involve higher-level information integration, whereas negative expectations sensitize the perception of discomfort via priming of early nociceptive activations through which the somatosensory input is processed.

## Results

### Task design

**Fig 1:**
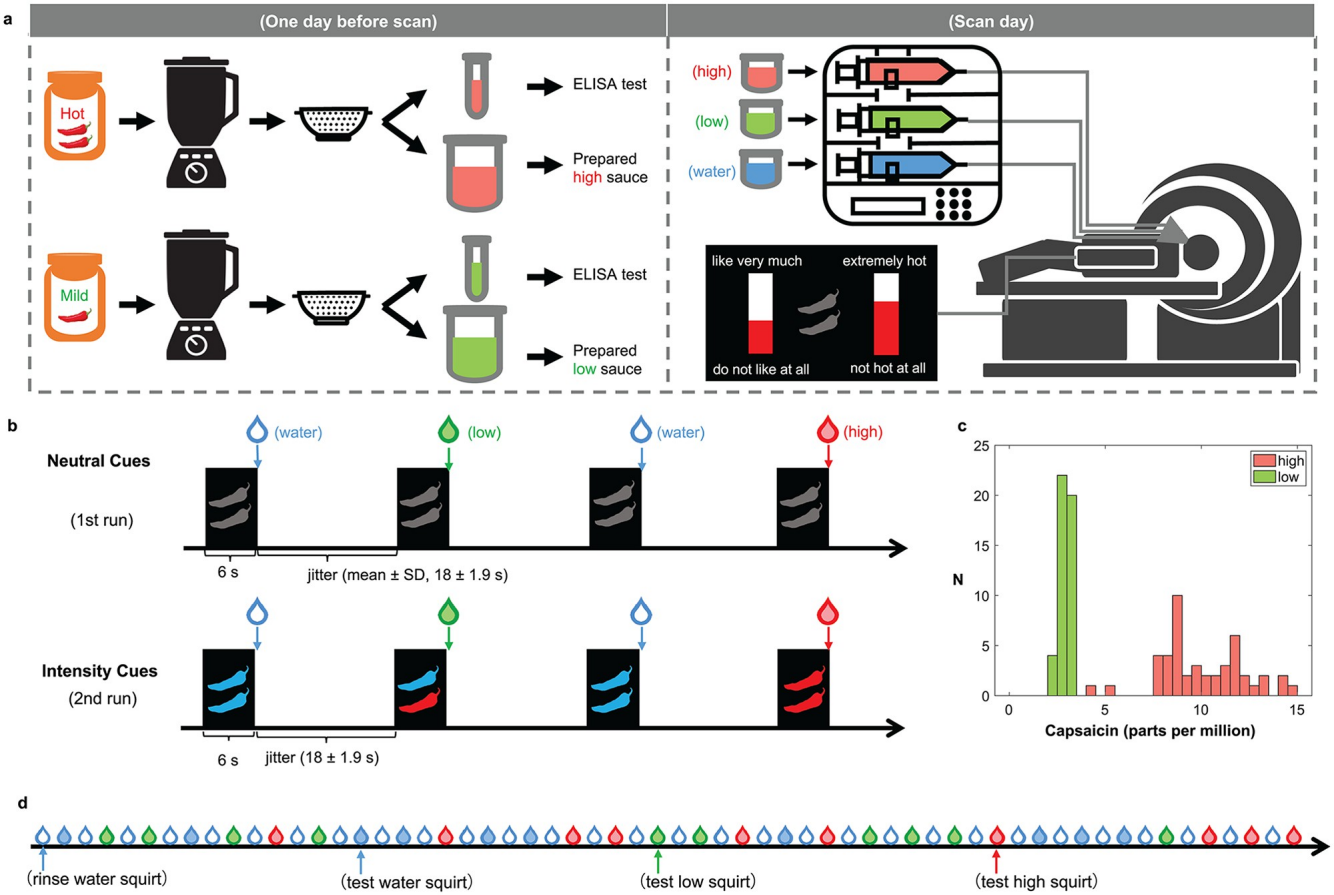
The design and stimuli of the task. (**a**) One day before the scan, high- and low-intensity hot sauces were prepared (i.e., blended and filtered to remove the pulp from the sauce). On the scan day, squirts of high-intensity hot sauce (high sauce), low-intensity hot sauce (low sauce), and water were delivered into the participant’s mouth while they lie in the scanner. Participants rated their experiences at any time during the task on 2 dimensions: experienced heat and flavor preference of the taste in their mouth. (**b**) Each participant completed 2 runs in the scanner. In the first run with *Neutral Cues*, 2 gray chili peppers were displayed 6 s before each squirt was delivered. In the second run with *Intensity Cues*, 2 colored chili peppers were displayed 6 s before squirt delivery. The color of the peppers indicates the spiciness of the squirts—2 red peppers are always paired with high sauce, 1 red and 1 blue peppers are always paired with low sauce, and 2 blue peppers are always paired with water and rinse. The interval between any squirt and its following cue was jittered (mean ± SD, 18 ± 1.9 s). (**c**) As confirmed by ELISA tests, the concentration of capsaicin in the high sauce was significantly higher than that in the low sauce. (**d**) An example sequence of 60 squirts in 1 run. Each run includes 30 test squirts (10 water, 10 low, and 10 high in a randomized order) and 30 rinse squirts, each preceding a test squirt.

Participants undergoing fMRI scanning tasted squirts of high- and low-intensity hot sauces, as well as squirts of water (Fig 1). They were asked to continuously rate their experiences during the task on 2 dimensions: sensory experience (“How ‘spicy’ or ‘hot’ does your mouth feel”) and hedonic experience (“How much do you like the flavor that you taste?”; Fig 1a). Each participant completed 2 runs in the scanner (Fig 1b). In the first run with *Neutral Cues*, 2 gray chili peppers were displayed 6 s before each squirt was delivered. This provided no information about the intensity or quality of the squirt to be delivered. The second run contained *Intensity Cues*; the same chili pepper shapes were colored to indicate the spiciness of the squirts 6 s before squirt delivery. Specifically, 2 red peppers are always paired with high-intensity hot sauce (high sauce), 1 red and 1 blue peppers are always paired with low-intensity hot sauce (low sauce), and 2 blue peppers are always paired with water. As shown in Fig 1c, the concentration of capsaicin in the high sauce (mean ± standard error: 10.06 ± 0.29 parts per million [PPM]) was significantly higher than that in the low sauce (2.89 ± 0.04 PPM), *t*(46) = 25.9, *p* < 0.001, *Cohen’s d* = 3.78.

Presenting the neutral cue run before the intensity cue run was strategically chosen to establish a baseline response to the neutral cue before introducing the potential interference or contamination from intensity cues. This approach allowed us to probe the participants’ preferences before the potential inference of expectation manipulation.

To increase the dynamics of changes in taste sensations during the task, we implemented a rinsing protocol. Before each of the 30 test squirts in both runs, participants received a rinse squirt of water (Fig 1d as an example). This procedure helps participants rinse their mouths, and reduce potential spiciness stimulation, back to a baseline level, thereby mitigating any potential adaptation effects.

We first pooled all participants (*n* = 47) together as 1 group (details in S1 Text) and found that self-reported spiciness was modulated by informative cues (S1 Fig). This effect was accompanied by increased activations during sauce tasting in brain regions long associated with pain processing and placebo effect—the bilateral anterior insula (AI) and DLPFC [22] (S2 Fig; see details in S2 Text).

Participants were recruited without advertising that the research was investigating “spicy” sauces. Once consented for a “sauce tasting” experiment, participants were told that the sauces may or may not be spicy; they were told that sensory experience may be pleasant or unpleasant according to their preference for spiciness, and then had the option to opt out if concerned. Despite this disclaimer, we were able to recruit comparable groups of participants who disliked (*n* = 22) and liked (*n* = 24) spicy sauces.

### Results differentiating participants who like and dislike spicy taste

Large individual differences in preference for the tasted sauces were demonstrated by the correlation of sensory ratings and hedonic ratings of each participant (S3 Fig and S1 Table). Some participants showed positive correlations between their hedonic and sensory ratings, with higher heat intensity ratings associated with higher pleasure ratings. Others showed negative correlations, with increased intensity of spiciness associated with decreased pleasure. To better understand the interaction between sensory experiences and subjective preferences, we separated participants who “liked” from those who “disliked” the spicy taste according to the sign of the correlation coefficients (S1 Table). This enabled us to dissociate the effects of positive expectations (expecting spicy squirts in the liking group) and negative expectations (expecting spicy squirts in the disliking group) while keeping sensory expectations constant across the 2 groups (expectations about receiving a high or low sauce). Indeed, the heat intensity ratings of high sauces (and low sauces) were similar across groups during the *Neutral cue* conditions (High sauces: Liking group [mean ± SD]: 1.55 ± 0.74; disliking group, 1.72 ± 0.85; *p* = 0.466. Low sauces: Liking group: 0.35 ± 0.72; disliking group, 0.38 ± 0.57; *p* = 0.836). However, visual *Intensity Cues*, which generated expectations about the upcoming sauce squirt, changed reported experiences.

#### Positive expectations decrease reported sensory experience of spiciness and increase associated neural responses

**Fig 2:**
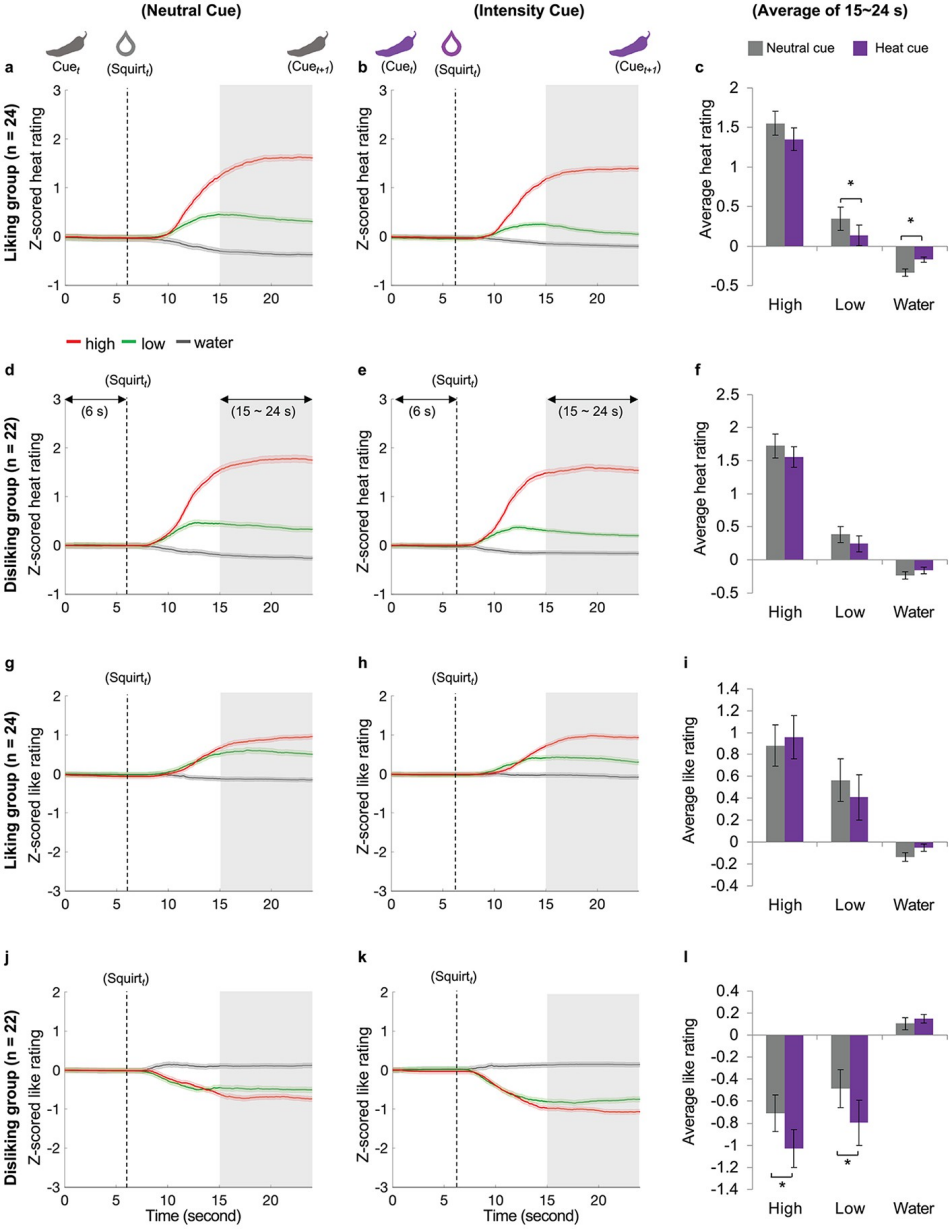
The reported spiciness and preference in the liking and disliking groups. Top 2 rows: heat intensity ratings; bottom 2 rows: like ratings. (**a, b**) The average ratings of spiciness with *Neutral Cue* (a) and those with *Intensity Cue* (b) for the liking group. Solid lines indicate the average across all trials in all subjects. The shaded area indicates the standard error of mean. (**d**, **e**) The average ratings of spiciness with *Neutral Cue* (d) and those with *Intensity Cue* (e) for the disliking group. (**c**, **f**) The heat ratings averaged over 15 to 24 s after cue display for the liking group (c) and the disliking group (f). (**g**, **h**) The average rating of preference with *Neutral Cue* (g) and those with *Intensity Cue* (h) for the liking group. (**j**, **k**). The average ratings of preference with *Neutral Cue* (j) and those with *Intensity Cue* (k) for the disliking group. (**i**, **l**) The like ratings averaged over 15 to 24 s after cue display for the liking group (i) and the disliking group (l). *Bonferroni corrected *p* < 0.05. Error bars in **c, f, j, and I** indicate standard error. Individual data are deposited in https://osf.io/cvjtd/?view_only=82aa9d97102c425f963ab1b4e52e8580.

Although the interaction of group (liking group, disliking group) × stimulus (high, low, water) × expectation manipulation (neutral cue, intensity cue) for the heat rating was not significant, *F*(2,88) = 0.42, *p* = 0.657, *η*^*2*^_*p*_ = 0.010, we conducted planned comparisons within the liking group and the disliking group respectively. In the liking group (first row in Fig 2; *n* = 24), the interaction between stimulus and expectation manipulation for the heat rating was significant, *F*(2,46) = 6.49, *p* = 0.003,$\eta_{p}^{2}=0.220$. During the saturation phase (15 s to 24 s post cue display), the liking group rated the low sauce associated with *Intensity Cues* (0.13 ± 0.63) as less spicy than the same sauce associated with *Neutral Cues* (0.34 ± 0.72), *p* = 0.002, whereas heat ratings for water after Intensity Cue (−0.17 ± 0.18) was rated as closer to the baseline than water after *Neutral Cue* (−0.34 ± 0.23), *p* = 0.002. This group’s heat intensity rating for the high sauce with *Intensity Cues* (1.35 ± 0.70) and those with *Neutral Cues* (1.55 ± 0.74) were not significantly different, *p* = 0.089. These findings suggest that positive expectations may modulate the perceived spiciness of low sauces more effectively than high sauces in individuals who like spicy foods. In contrast, in the disliking group (second row in Fig 2), the interaction between stimulus and expectation manipulation for the heat rating was not significant, *F*(2,42) = 1.69, *p* = 0.196,$\eta_{p}^{2}=0.075$. Individual **behavioral rating trajectories are shown in** S4 Fig.

**Fig 3:**
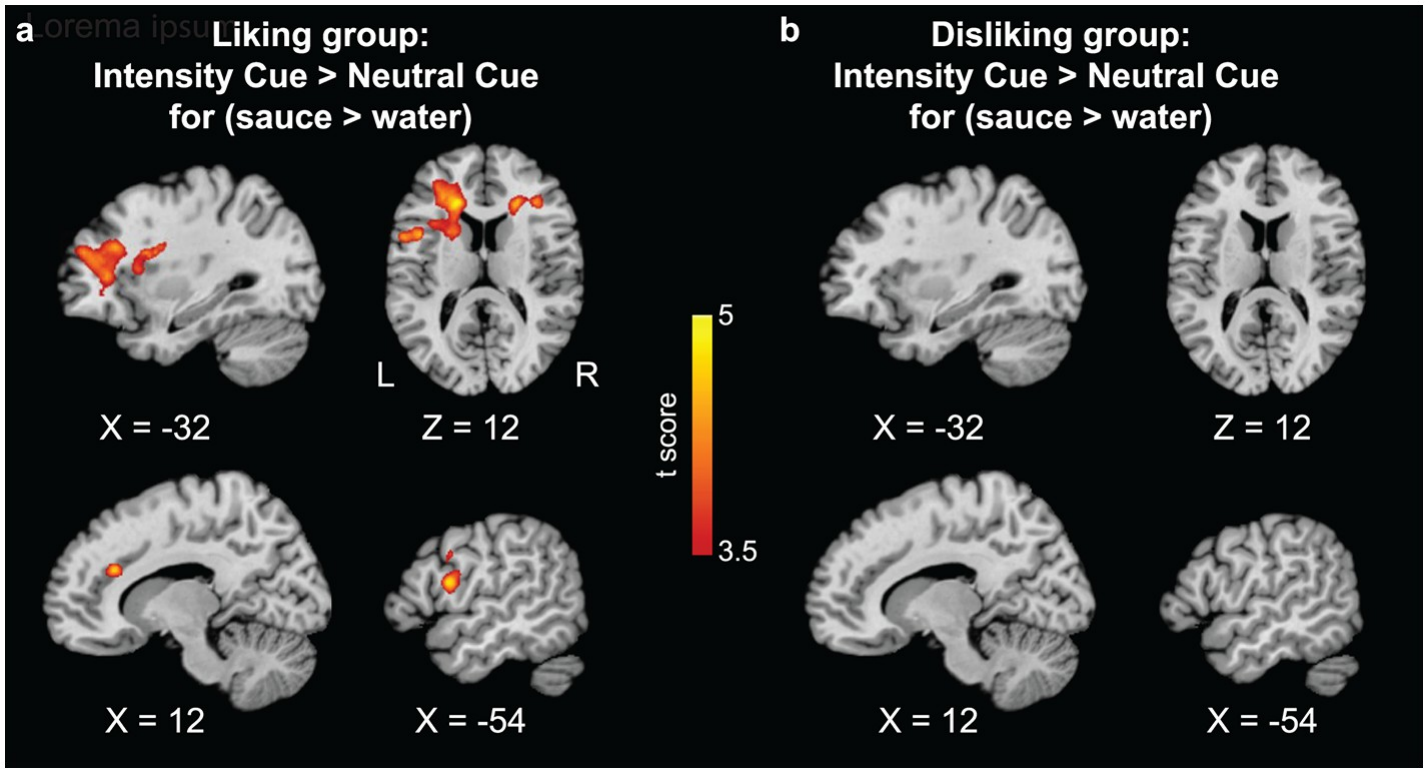
Brain responses for the interaction of *Intensity Cue* (sauce > water) > *Neutral Cue* (sauce > water). (**a**) Stronger brain responses for the liking group for sauce than water when information of spiciness was presented. (**b**) For the contrast of sauce > water, the disliking group did not show different brain response between *Intensity Cue* and *Neutral Cue* conditions. FWE cluster-wise corrected, *p* < 0.05, cluster-defining threshold *p* < 0.001.

In terms of neural responses as measured by fMRI, only the liking group showed a significant effect for the contrast of *Intensity Cued* greater than *Neutral Cued* sauce-tasting (high and low sauces pooled) versus water. Stronger BOLD responses in the left AI, left DLPFC, and right dorsal ACC were observed when tasting sauces paired with *Intensity Cues* compared to tasting sauces that were paired with *Neutral Cues* (Fig 3a and S2 Table). In the disliking group, the interaction between stimulus and expectation manipulation was not significant for any cluster. To account for the hemodynamic responses starting at 15 s after cue display (i.e., the saturated phase), we conducted an additional analysis that included an additional regressor in the first-level GLMs, with an onset at the start of the saturated phase and a duration of 9 s (details in S1 Text). As shown in S5 Fig, this new analysis demonstrates that the pattern of brain response to squirt delivery remains consistent with our original analysis (Fig 3), while no significant findings were observed for the new regressor representing the saturated phase brain response. These results indicate that the pattern we found in brain activation is robust. In line with the behavioral finding, these results suggest that brain responses to such top-down modulation of sensory intensity processing predominantly manifest in response to positive expectations. A further analysis separating high and low sauces found that the effect of low sauce (S6c and S6d Fig) was consistent with the pattern when high and low were pooled. This aligns with our behavioral results that positive expectations in the liking group led to a significant decrease in the perceived spiciness of the low sauce, but not the high sauce. This suggests that the modulation effect of positive expectations could be more pronounced for milder stimuli in individuals who like spicy tastes.

Interestingly, the response to squirts in the pleasure signature identified by a previous meta-analysis [23] in the liking group also displayed a significant interaction between stimulus and expectation manipulation, *F*(2,40) = 3.44, *p* = 0.042,$\eta_{p}^{2}=0.147$. Consistent with our findings in heat ratings, only when the *heat intensity* was cued, this group had stronger pleasure-signature activations for low sauce (mean ± standard error: 0.64 ± 0.41) than water (−1.16 ± 0.34), *p*_*uncorrected*_ = 0.044 (S7 Fig). In contrast, the disliking group showed no significant interaction between the stimulus and expectation manipulation, *F*(2,36) = 2.197, *p* = 0.126,$\eta_{p}^{2}=0.109$. This result suggests that positive expectations are more likely to modulate pleasure-related neural responses, echoing previous hypotheses linking placebo expectations and reward [16,24].

#### Negative expectations increase anhedonic experience of spiciness and elevate NPS response

The interaction of group × stimulus × expectation manipulation for the like rating was not significant, *F*(2,88) = 1.71, *p* = 0.187, *η*^*2*^_*p*_ = 0.037. However, as revealed by our planned comparisons, in the disliking group (fourth row in Fig 2; *n* = 22), the interaction between stimulus and expectation manipulation for the preference rating was significant, *F*(2,42) = 3.678, *p* = 0.034,$\eta_{p}^{2}=0.149$. The disliking group rated the high (mean ± SD: −1.03 ± 0.81) and low (−0.80 ± 0.97) sauces after *Intensity Cues* as less favorable than those after *Neutral Cues* (high: −0.71 ± 0.79, *p* = 0.036; low: −0.49 ± 0.80, *p* = 0.013), while liking ratings for water after *Intensity Cues* (0.15 ± 0.18) and water after *Neutral Cues* (0.10 ± 0.26) were not different, *p* = 0.459. By contrast, in the liking group (third row in Fig 2), the interaction between stimulus and expectation manipulation for the liking rating was not significant, *F*(2,46) = 2.396, *p* = 0.102,$\eta_{p}^{2}=0.094$. These results demonstrate that the hedonic experiences of tasting spicy sauces were modulated by negative expectations but not positive expectations.

**Fig 4:**
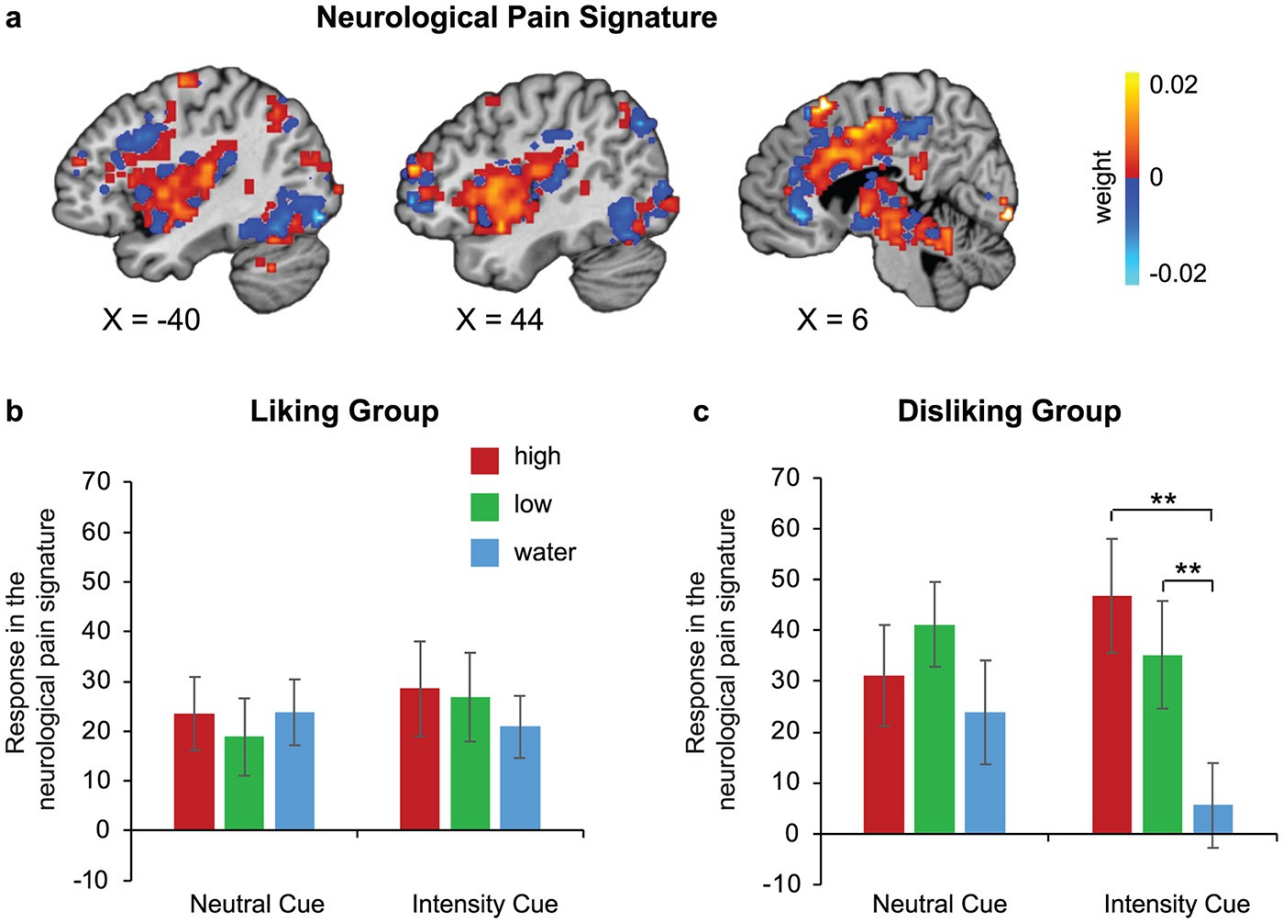
Responses in the neurological pain signature in participants grouped by the intrinsic preference for the spicy taste. (**a**) The neurological pain signature (NPS, [25]) applied to each participant’s first-level general linear model through a linear combination defined by the weights. A scalar value for each participant was obtained as the response in the NPS which indicates predicted pain intensity. (**b**) The responses to squirts in the neurological pain signature for the liking group. (**c**) The responses to squirts in the NPS for the disliking group. Only with *Intensity Cue*, this group had stronger activations for sauce than water. ** Bonferroni corrected *p* < 0.001. Individual data for **b** and **c** are deposited in https://osf.io/cvjtd/?view_only=82aa9d97102c425f963ab1b4e52e8580.

In the disliking group, the responses of the NPS [25] to squirts demonstrated a significant interaction between stimulus and expectation manipulation, *F*(2,36) = 5.066, *p* = 0.012,$\eta_{p}^{2}=0.220$. Only when the *heat intensity* was cued, this group had stronger NPS activations for high (mean ± standard error: 46.73 ± 11.23) and low (35.21 ± 10.58) sauces versus water (5.56 ± 8.38). By contrast, the liking group showed no significant interaction between the stimulus and expectation manipulation, *F*(2,40) = 0.942, *p* = 0.398, $\eta_{p}^{2}=0.045$(Fig 4). Since NPS is a validated measure that tracks levels of nociceptive pain [26,27], this finding indicates that the modulation of heat intensity and the anhedonic experience associated with negative expectations might manifest via priming of nociceptive processes.

We further explored whether the sensory or the affective aspect of the NPS was more likely modulated by the negative expectations in our task. We conducted ROI analyses within each of the key regions: S1 and thalamus (sensory); insula and amygdala (affective). We found a significant interaction between stimulus and expectation manipulation in the response in insula to squirts, *F*(2,36) = 4.30, *p* = 0.021,$\eta_{p}^{2}=0.193$. Only when the heat intensity was cued, this group had stronger insula activations for high (1.61 ± 1.37, *p* = 0.026) and low (1.61 ± 1.71, *p* = 0.005) sauces versus water (0.47 ± 0.43). Since the insula has been associated with negative emotion processing, this result suggests that negative expectations enhanced the affective aspect of pain processing in the disliking group. In comparison, the activations in amygdala and the sensory-related ROIs (S1 and thalamus) did not show an interaction of stimulus and expectation manipulation. This suggests that the observed modulation of the NPS by negative expectations might be primarily driven by changes in the emotional rather than the sensory component of pain processing.

## Discussion

By controlling the intensity of sensory input and pairing these stimuli with visual cues, we found that the subjective experience of spiciness, as well as activations in AI and DLPFC, were modulated by expectations about the intensity of spiciness. Furthermore, by differentiating positive and negative expectations based on individual differences in hedonic preference for spicy taste, we demonstrate that, when the sensory expectations remained the same, positive expectations decreased the reported heat intensity and increased responses in AI, dorsal ACC, and DLPFC, as well as a predefined pleasure signature [23], while negative expectations decreased reported preference for the tasting experience and elevated a neural pain signature (NPS) response. Together, our results show that even when the sensory experience is the same, hedonic expectations modulate people’s conscious reports of their subjective experiences. Positive expectations appear to engage pleasure processing and neural processes that promote information integration over sensory experiences, whereas negative expectations appear to influence earlier nociceptive or negative affective processes.

The effect of expectation has been observed in previous studies with different types of stimuli (e.g., beverages [28], wine [29], thermal pain [30], and nicotine [31]). Here, we found that when pooled together, participants’ subjective experiences of capsaicin spiciness changed by merely presenting visual cues indicating how intense the sensory input would be. Further, AI and DLPFC were sensitive to this expectation manipulation—when tasting the expected sauces, both regions had elevated activations compared to the same sauces being tasted without the intensity predictively cued. AI has been proposed to integrate sensory and visceral information to reach an interoceptive representation of the body that guides decision-making [31]. DLPFC has been associated with integration processes that support both perceptual and value-based decision-making [32,33]. Consistent with these prior works, our results suggest that the expectations triggered by visual cues were integrated with the actual somatosensory input to generate significant changes in participants’ subjective experiences.

Importantly, our findings provide novel insight over previous observations into the influence of expectations on subjective states and preferences [20,34,35] by dissociating the behavioral and neural mechanisms underlying the effects of expectations about sensory versus hedonic value. By using a quantifiable sensory stimulus (i.e., capsaicin in a flavored sauce), we were able to deliver the same objective sensory expectation and stimulus while observing the effects of polar opposite expectations of hedonic value.

On the one hand, in people who preferred the spicy sauces, the subjective experience of spiciness intensity changed after simply observing a predictive visual cue that generated an expectation of capsaicin intensity. These expectations also induced increased activation in the AI, dorsal ACC, DLPFC, and areas in the pleasure signature for spicy sauces, but only in the group that liked the spicy sauces. These findings suggest a specific neural effect of positive expectations. Psychophysiological changes driven by positive expectations have been proposed to be a key underlying mechanism for placebo effects [5] and were linked to reward anticipating/processing [24]. Indeed, previous studies in the placebo research field also found that open administration (expecting a positive treatment outcome) of medical treatments is more effective than hidden administration (no expectation) [36,37].

The increased activations in dorsal ACC and DLPFC induced by positive expectation echo the result from a recent meta-analysis identifying increased DLPFC and ACC responses associated with placebo effect [9]. Previous studies found that the disruption of the function of bilateral DLPFC [38], as well as the connectivity between DLPFC and ACC [39,40] could block or attenuate placebo effects, suggesting a causal role of these regions in the impact of expectation on perception and subjective states. The DLPFC and ACC have been related to core executive functions that generate, integrate, and modulate cognitive representations needed to produce the contextually appropriate response [41,42]. Meanwhile, the significant differences observed in ratings between the neutral and intensity cue conditions, along with the lack of significant differences in reaction times for heat ratings across runs (S3 Table), suggest that potential cognitive load difference between runs did not differentially affect the participants’ heat ratings. The AI has also been considered important for the integration of external environment and internal state and generates subjective feelings that guide behavior [43,44]. Taken together, these studies demonstrate that the modulation of positive expectation involves complex affective and cognitive processes that evaluate and integrate contextual cues and sensory input as well as their internal states.

On the other hand, in people who did not like the spicy sauces, visual cues that predicted the spiciness intensity did not change their subjective rating of capsaicin intensity, but these cues did significantly decrease their rating of the pleasantness of the sauce tasting compared to the same sauces tasting without intensity-predicting cues. This behavioral finding suggests a specific exacerbation of discomfort induced by negative expectations. However, we should hasten to note that while we observed distinct effects within the liking group and disliking group, respectively, these should not be interpreted as differences between the groups in the absence of significant interaction effects. Interestingly, negative expectations also induced stronger NPS responses, a validated measure that tracks levels of nociceptive pain [26,27]. Thus, modulation of spiciness by negative expectations might have sensitized these participants to the perception of discomfort and even pain [35,45], via priming of early nociceptive activations through which the somatosensory input was processed. A recent meta-analysis found that the placebo effect was modest on the NPS [17]. As a comparison, our findings suggest that, unlike the effect of positive expectations that support the placebo effect and engage pleasure processing, negative expectations enhance early nociceptive processes, indicating asymmetric effects of the 2 types of expectations. Future studies using temporally more specific tools such as magnetoencephalography or electroencephalogram could help determine whether the influence of negative expectations happens earlier than the effect of positive expectations.

Our findings indicate an asymmetric effect of hedonic expectation on sensory experience. Specifically, positive expectations (intensity cues) led to a significant reduction in the perceived spiciness of the low sauce in the liking group. This modulation was not observed in the disliking group or for high sauces in either group. The lack of significant interaction for high sauces may suggest a ceiling effect, where the high baseline intensity of spiciness might have limited the modulatory impact of expectations. This highlights the nuanced nature of sensory modulation by expectations, indicating that positive expectations may only significantly alter sensory perception when the stimulus intensity is not overwhelming. This asymmetric effect underscores the influence of hedonic predisposition on how sensory expectations are processed and perceived.

In summary, the current study brings novel insights into the interplay of psychological and neural processes underlying the interaction between mind, body, and environment. Our study contributes to the growing body of literature on the mechanisms of hedonic expectations by demonstrating neural and behavioral responses to positive and negative expectations. Earlier studies that induced positive or negative expectations showed modulated sensory and emotional responses [46–48], our experimental design provides a more comprehensive assessment of both sensory and hedonic responses to pain, with hedonic expectation and sensory expectation dissociated. In alignment with recent meta-analytic evidence showing that placebo analgesia is associated with higher-level cognitive and affective processes [49] and that distinct neural circuits are involved in placebo and nocebo effects [14], we further demonstrate that when sensory expectations are kept consistent, the hedonic characteristic of one’s expectations modulates their experiences, as well as the associated neural processes in an asymmetric manner: positive expectations modulate the intensity of subjective experience and engage brain areas involved in information integration and reward processing, while negative expectations diminish hedonic experience and elevate neural activity regions associated with pain processing, particularly its affective component. This nuanced understanding of top-down modulation of sensory experience offers potential avenues for the development of tailored interventions that harness the power of expectations for improved patient care and treatment efficacy in clinical settings.

## Methods

### Ethics statement

The study has been conducted according to the principles expressed in the Declaration of Helsinki. All participants gave written consent to participate in the study, and all procedures were approved by Virginia Tech Institutional Review Board (approval number: VT IRB# 11–377).

### Participants

Fifty-three participants enrolled in the current study. Among them, 48 participants completed the 2 runs of tasks while undergoing fMRI scanning. The other 5 participants who only completed the first run of the task were excluded from data analyses. One participant was excluded because they did not respond behaviorally in the second run of the task, which leaves 47 subjects in total who completed the experiment (23 females, 30 ± 11.4 years old [mean ± SD]). Finally, only 1 participant did not show a significant correlation (positive or negative) between their heat intensity and sauce preference ratings and was excluded from further analyses. None of the participants have a history of neurological or psychiatric disorders. All participants had a normal or corrected-to-normal vision.

### Stimuli

#### Hot sauce preparation and delivery

As shown in the left panel in Fig 1a, high sauce and low sauce were prepared for each participant 1 day before the scan. “*Arriba*! *hot red salsa*” sauce was blended and then filtered through a cheesecloth to remove the pulp from the sauce, which was then saved in the refrigerator to be used as the high sauce in the experiment. A small portion of the prepared sauce was saved in a separate test tube and sent to the lab for ELISA (enzyme-linked immunosorbent assay) test. The same procedure was done on “*Arriba*! *mild red salsa*” sauce to prepare the low sauce used in the experiment. ELISA tests to identify the concentration of capsaicin were done for high and low sauces used for each participant (details in S1 Text).

### Experiment procedure

On the scan day, while the participant was lying in the scanner, high and low sauces, as well as water were delivered into the participant’s mouth, one squirt at a time, through 3 tubes (one for each type of squirt) bundled by a mouthpiece (Fig 1a, right panel). Sauces and water were slowly ejected from the tubes using a computer-controlled syringe pump (Harvard Apparatus) located outside of the scanner room. At any time during the task, participants were to rate their subjective experiences about the current taste in their mouth on 2 scales which were discretized in 11 arbitrary units on a vertical visual digital scale (i.e., ranging from 0 to 10, numbers not shown on scale, Fig 1a). We asked the participants to rate 2 dimensions of subjective experiences throughout the task: (1) “How ‘spicy’ or ‘hot’ does your mouth feel” (0 = not hot at all; 10 = extremely hot), and (2) “How much do you like the flavor that you taste?” (0 = do not like at all; 10 = like very much) (detailed instructions in S1 Text). Throughout the entire task, 2 rating bars were presented side-by-side—one to rate “how hot” the other to rate “preference.” All ratings were made through hand-held button boxes. The side that each rating bar was presented (and hand used to control rating) was randomized at the level of participant, so that one participant would always rate “heat” with the same hand and “preference” with the other but across participants, the hand used to rate each scale was counterbalanced. Specifically, participants were asked to continuously update their subjective experiences by adjusting the rating bar to the appropriate level based on how the experience in their mouth evolved. We stressed that the participants need to adjust these bars as the experience changed, not just when they receive a squirt and swallow, but throughout the entire task (e.g., while they receive a squirt, while they taste the sauce, during and after they have swallowed, and while they wait for the next squirt). Throughout the experiment, participants could raise or lower these bars to whatever levels they feel best described their level of experience as the experience changed. Stimuli were presented and participants’ behavioral responses were collected using NEMO (Human Neuroimaging Laboratory, Fralin Biomedical Research Institute at VTC, Virginia Tech).

#### Run 1 with *Neutral Cues*

In the first run, a total of 60 squirts of sauce or water were delivered into the participant’s mouth, one squirt at a time. Among them, 30 test squirts, including 10 water squirts, 10 low squirts, and 10 high squirts, were delivered in a randomized order. In addition, 1 water squirt (a rinse squirt) was administered before each of the 30 test squirts to rinse and restore the participant’s mouth to a baseline level prior to the next test squirt (Fig 1d as an example). Six seconds prior to each squirt, a visual cue showing 2 gray peppers (i.e., *Neutral Cue*) was displayed (Fig 1b). Each cue was displayed for 6 s, and each squirt was delivered immediately after the cue disappeared. Each squirt was approximately 1 ml and delivered within 1 s. The interval between any squirt and its following cue was jittered (mean ± SD, 18 ± 1.9 s).

#### Run 2 with *Intensity Cues*

The second run was nearly identical to run 1, except that the visual pepper cues were colored to indicate the heat intensity of the upcoming squirt (Fig 1b). A total of 60 squirts were delivered in this run, including 30 test squirts (10 water, 10 low, and 10 high) in a randomized order and 30 rinse squirts, each preceding a test squirt. The interval between any squirt and its following cue was also jittered (mean ± SD, 18 ± 1.9 s). A cue with 2 colored peppers (*Intensity Cue*) indicated the intensity of the spiciness of the upcoming squirt displayed 6 s before each squirt to indicate its spiciness: 2 red peppers for “high” sauce, 1 red pepper and 1 blue pepper for “low” sauce, and 2 blue peppers for water and rinse. Participants were not explicitly informed of the meaning of the cues.

### Behavioral data analysis differentiating participants who liked versus disliked spicy taste

A Spearman’s rank-order correlation comparing the participant’s ratings of “heat” and “like” during run 1 (there were no expectations about sauce spiciness intensity cued) was conducted to determine their preference for the spicy sauces (see S1 Table for the correlation coefficient and significance of correlation for each participant). Participants with a positive correlation were operationally defined as participants who like spicy sauce (Liking group, *N* = 24, 11 females, 32 ± 11.6 years old), whereas those with a negative correlation were operationally defined as participants who dislike spicy sauce (Disliking group, *N* = 22; 12 females, 27 ± 10.9 years old). One participant did not show a significant correlation between the 2 subjective rating scales and was excluded from further analysis. Confirmed by independent-samples *t* tests, there was no significant difference between the Liking group and the Disliking group in the concentrations of capsaicin either in the high sauce (Liking group: 9.89 ± 0.42 PPM; Disliking group: 10.20 ± 0.42 PPM; *t*(44) = 0.514, *p* = 0.611, *Cohen’s d* = 0.152) or in the low sauce (Liking group: 2.92 ± 0.07 PPM; Disliking group: 2.87 ± 0.06 PPM; *t*(44) = 0.534, *p* = 0.596, *Cohen’s d* = 0.158).

#### Heat rating comparisons

For each participant, the subjective rating of experienced heat on a 0 to 10 scale (integers from 0 to 10) was converted to z-scores which made each participant’s heat ratings have a mean of 0 and standard deviation of 1 (see S8 Fig for raw heat ratings). The z-scored rating was interpolated with zero-order hold (ZOH) at 0.1 s intervals. In ZOH interpolation, the value of each sample is held constant until the next sample point is reached. This means that the signal is essentially represented by a series of constant segments between consecutive sample points, creating a staircase-like approximation of the original signal. A correction was then done with the baseline being the average rating during the 5 s before the cue display.

We first conducted a 3-way ANOVA test of group (between-subjects: Liking group, Disliking group) × stimulus (within-subjects: high, low, water) × expectation manipulation (within-subjects: *Neutral Cue*, *Intensity Cue*) on the average heat rating during the saturated phase (15~24 s after the cue displayed). After that, we conducted a repeated 2-way ANOVA test of stimulus (within-subjects: high, low, water) × expectation manipulation (within-subjects: *Neutral Cue*, *Intensity Cue*) was also done within each group on the average heat rating during the saturated phase. This planned comparison was based on well-established literature (e.g., [8,14,50,51]) that expectations can be associated with either positive or negative valence and exert different effects. Post hoc tests were done to confirm the effect of expectation on each type of squirt in each group.

#### Like rating comparisons

The subjective rating of flavor preference was also converted to z-scores within each subject. The z-scored heat ratings were interpolated with ZOH at 0.1 s intervals and then baseline-corrected by the average rating during the 5 s before the cue display.

We also conducted a 3-way ANOVA test of group × stimulus × expectation manipulation on the average like rating during the saturated phase. After that, a repeated 2-way ANOVA test of stimulus × expectation manipulation was also done within each group on the average like rating during the saturated phase, followed by post hoc tests checking the effect of expectation on each type of squirt in each group.

### fMRI data acquisition and preprocessing

Imaging was conducted on a 3.0 Tesla Siemens Trio scanner. High-resolution T1-weighted scans (voxel size: 0.479 × 0.479 × 1.0 mm) were acquired using an MPRAGE sequence (Siemens). Functional images were acquired using echoplanar imaging (repetition time = 2,000 ms; echo time = 30 ms; flip angle = 90°; 34 slices; voxel size: 3.44 × 3.44 × 4.0 mm). Images were analyzed using SPM12 (http://www.fil.ion.ucl.ac.uk/spm/software/spm12/). The first 2 EPI volumes of each run were discarded for signal stabilization. Slice timing correction was first applied to temporally align all the functional images with the temporal middle slice as the reference. Motion correction to the first functional image was performed using a six-parameter rigid-body transformation. T1-weighted images were coregistered to the average of the motion-corrected images and segmented into gray matter, white matter, and cerebral spinal fluid images to estimate the normalization parameters to the Montreal Neurological Institute (MNI) template. These transformation parameters were applied to the functional images for spatial normalization to the MNI space and interpolated to 2 × 2 × 2 mm voxels. Finally, the normalized functional images were smoothed with an 8 mm isotropic Gaussian kernel and then high-pass filtered (128 s width) in the temporal domain. Two participants (both from the disliking group) were excluded because of compromised quality of imaging data. Four participants (3 from the liking group and 1 from the disliking group) with excessive head motions were excluded from further analyses. Therefore, 21 participants from the liking group, 19 participants from the disliking group, and another participant who did not belong to either group were included in further brain-level analyses.

### fMRI data analyses

#### Univariate voxel-wise analysis of fMRI data

General linear models (GLMs) were specified for each participant (first-level analysis). All visually and orally delivered stimuli and motor responses were modeled in the design matrix that was constructed by convolving each event onset with a canonical hemodynamic response function in SPM12. Residual effects of head motion were corrected by including the estimated 6 motion parameters for each participant as covariates. Low-frequency drifts were removed using a high-pass filter (128 s cutoff).

Beta maps at squirt delivery were estimated respectively for each regressor at each run for each participant and then entered into group-level analyses. After dividing participants into the liking group and the disliking group, we tested how positive and negative expectations manipulate brain responses to spicy taste by conducting a whole-brain contrast: (sauce__*Intensity Cue*_*−water*_*_ Intensity Cue*_*)–(sauce*__*Neutral Cue*_ versus water__*Neutral Cue*_) with high and low sauces pooled within each group. Additionally, we also tested the contrast of *(high*__*Intensity Cue*_*−water*_*_ Intensity Cue*_*)–(high*__*Neutral Cue*_ vs. water__*Neutral Cue*_) as well as the contrast of *(low*__*Intensity Cue*_*−water*_*_ Intensity Cue*_*)–(low*__*Neutral Cue*_ vs. water__*Neutral Cue*_) within each group. Statistics maps were overlaid on a standard brain in Montreal Neurological Institute space using Mango (http://ric.uthscsa.edu/mango/mango.html). The threshold for whole-brain analyses was set at *p* < 0.05, familywise error (FWE) cluster-wise corrected (cluster-defining threshold, *p* < 0.001) unless otherwise stated.

#### Pleasure signature response

To examine the activations in pleasure/reward-related brain areas, we conducted a region of interest (ROI) analysis using a mask of pleasure signature identified by a previous meta-analysis [23]. We extracted and averaged beta values from this mask, including only voxels with positive coefficients (i.e., positively predicting pleasure). To determine whether the pleasure signature response was specifically sensitive to positive expectation, we performed a three-way ANOVA test of stimulus (sauce, water) × expectation manipulation (*Intensity Cue*, *Neutral Cue*) × group (liking group, disliking group), followed by post hoc tests comparing responses to squirts in each Cue condition within each group.

#### Neurological pain signature response

To quantify the NPS response, the signature response was estimated for each test subject by taking the dot product of first-level activation images with the signature pattern, yielding a continuous scalar value. From there, to test whether NPS response was specifically sensitive to negative expectation, we ran a three-way ANOVA test of stimulus (sauce, water) × expectation manipulation (*Intensity Cue*, *Neutral Cue*) × group (liking group, disliking group), followed by post hoc tests comparing NPS responses to squirts in each Cue condition within each group. To further explore whether the sensory or the affective components of the NPS were more likely modulated by the negative expectations, we conducted ROI analyses within each of the key regions: S1 and thalamus (sensory), insula and amygdala (affective). Beta values were extracted from each of these regions defined by the automated anatomical atlas 3 [52] and then averaged. The same ANOVA and post hoc tests were done for these ROIs.

### Statistics

Statistics on behavioral data were implemented using IBM SPSS Statistics 21.0 (IBM Corp., Armonk, NY). The significance level was set at 0.05 for all analyses and Greenhouse–Geisser correction non-sphericity was used when appropriate. Post hoc comparisons were evaluated using two-tailed pairwise tests with Bonferroni correction. Partial eta-squared ($\eta_{p}^{2}$) and *Cohen’s d* values were provided to demonstrate effect size where appropriate.

## Supporting information

S1 Fig Experienced heat was modulated by merely providing information on spiciness. Individual data are deposited in https://osf.io/cvjtd/?view_only=82aa9d97102c425f963ab1b4e52e8580.
(DOCX)

S2 Fig Stronger brain responses for all participants pooled for sauce than water at squirt delivery. (DOCX)

S3 Fig Like ratings for each participant between heat and like ratings in Neutral Cue condition. (DOCX)

S4 Fig Individual behavioral rating trajectories. (DOCX)

S5 Fig Brain responses for the interaction of *Intensity Cue* (sauce > water) > *Neutral Cue* (sauce > water) at squirt delivery with saturated phase included in the first-level general linear model. (DOCX)

S6 Fig Brain responses for the interaction of *Intensity Cue (sauce > water) > Neutral Cue (sauce > water)* at squirt delivery in each group, with high- and low-intensity hot sauces analyzed separately. (DOCX)

S7 Fig Responses in the pleasant signature in participants grouped by the intrinsic preference for the spicy taste. Individual data for b and c are deposited in https://osf.io/cvjtd/?view_only=82aa9d97102c425f963ab1b4e52e8580.
(DOCX)

S8 Fig Raw heat ratings for each participant. (DOCX)

S1 Table Correlation coefficient and significance of heat and liking ratings in *Neutral Cue* condition for each participant. (DOCX)

S2 Table Clusters with stronger activations for sauce > water with *Intensity Cues* than *Neutral Cues*. (DOCX)

S3 Table Reaction time in each condition. (DOCX)

S1 Text Supplementary methods. (DOCX)

S2 Text Supplementary results. (DOCX)

## Abbreviations

ACC: anterior cingulate cortex
AI: anterior insula
DLPFC: dorsolateral prefrontal cortex
DPMS: descending pain modulatory system
ELISA: enzyme-linked immunosorbent assay
fMRI: functional magnetic resonance imaging
FWE: familywise error
GLM: general linear model
NPS: neurologic pain signature
PPM: parts per million
ROI: region of interest
ZOH: zero-order hold
