# Supplementary material for: The expectations humans have of a pleasurable sensation asymmetrically shape neuronal responses and subjective experiences to hot sauce
Source: PLoS Biol. 2024 Oct 8;22(10):e3002818. doi: 10.1371/journal.pbio.3002818 (PMC11460714; doi:10.1371/journal.pbio.3002818)
Supplement: S1 Fig — Individual data are deposited in https://osf.io/cvjtd/?view_only=82aa9d97102c425f963ab1b4e52e8580. (DOCX) [file pbio.3002818.s001.docx]

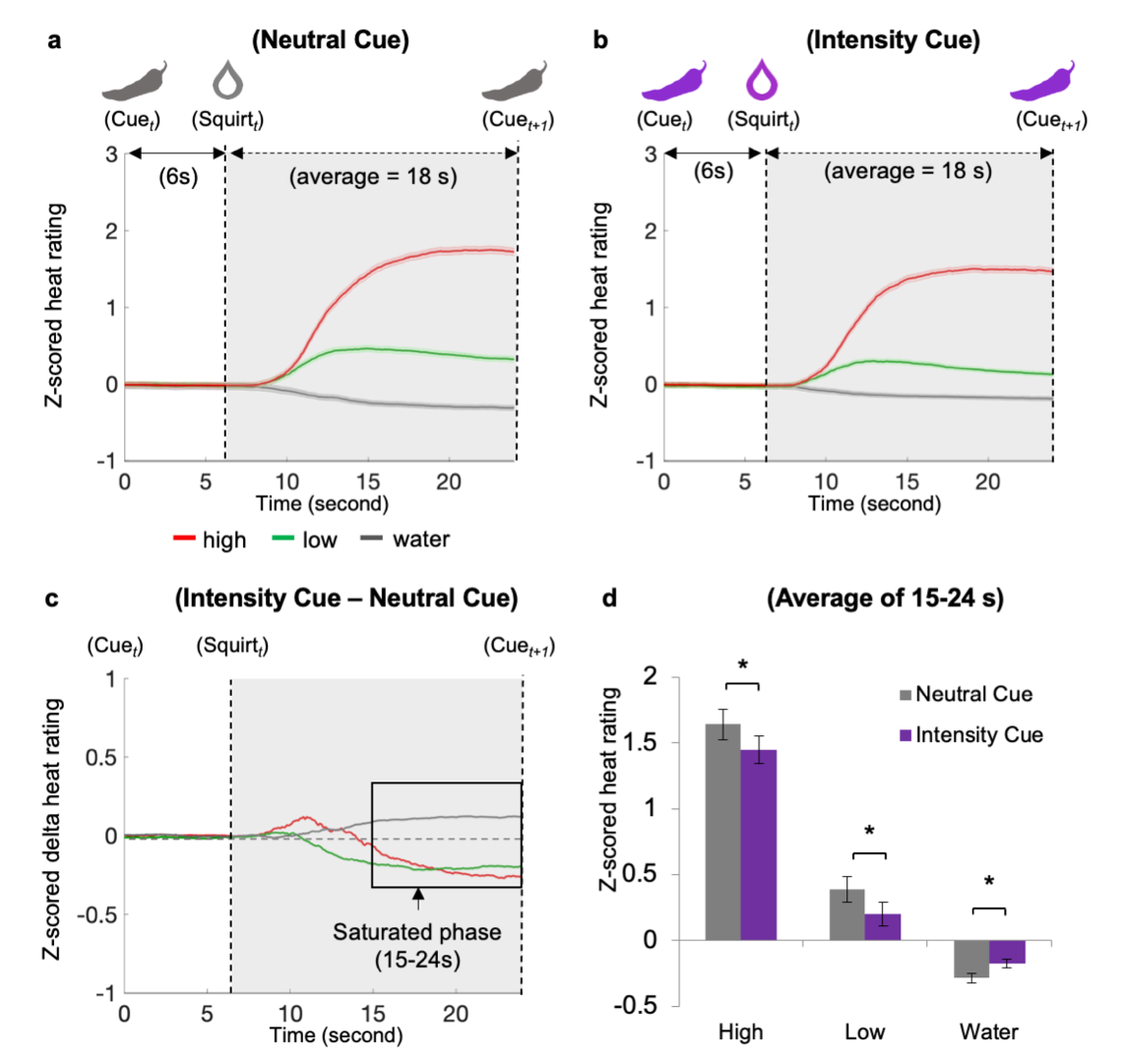


**S1 Fig**. Experienced heat was modulated by merely providing information on spiciness. **a & b.** The average ratings of experienced heat when neutral cues were provided (a) and those when visual *Intensity Cues* were provided (b). Solid lines indicate the average across all trials in all subjects. The shaded area indicates the standard error of mean. **c.** The average rating of experienced heat when *Intensity Cues* were provided was subtracted by that with *Neutral Cues* for each type of sauce or water. **d**. The heat ratings averaged across the saturated phase (15 to 24 seconds after cue display). High- and low-intensity hot sauces with *Intensity Cues* were rated as less spicy than that after *Neutral Cue*, whereas water with *Intensity Cue* was rated as significantly spicier than that with *Neutral Cue*. **p* < 0.05. Error bars indicate standard error. Individual data are deposited in https://osf.io/cvjtd/?view_only=82aa9d97102c425f963ab1b4e52e8580.
