# Supplementary material for: The expectations humans have of a pleasurable sensation asymmetrically shape neuronal responses and subjective experiences to hot sauce
Source: PLoS Biol. 2024 Oct 8;22(10):e3002818. doi: 10.1371/journal.pbio.3002818 (PMC11460714; doi:10.1371/journal.pbio.3002818)
Supplement: S2 Fig — (DOCX) [file pbio.3002818.s002.docx]

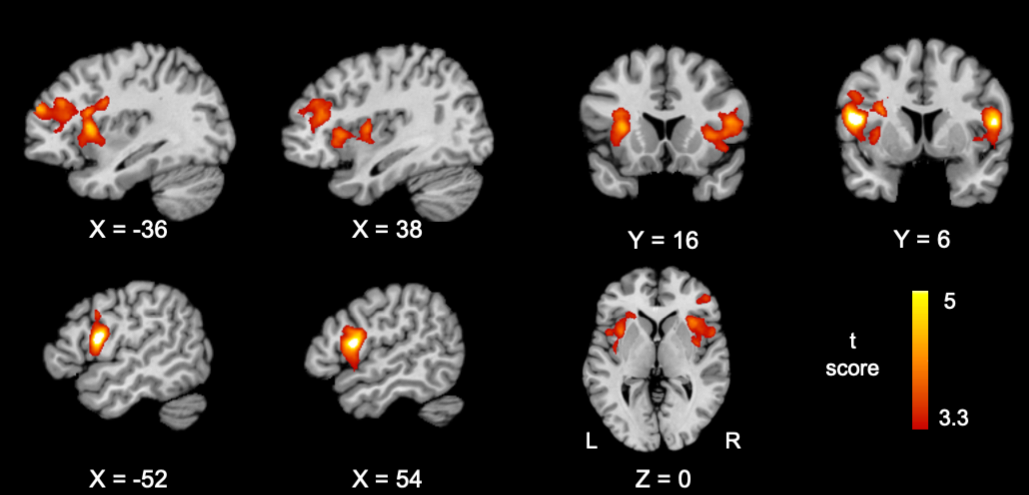


**S2 Fig**. Stronger brain responses for all participants pooled for sauce than water at squirt delivery. In the contrast of sauce > water, squirts with *Intensity Cue* induced stronger activations in the bilateral anterior insula and dorsolateral prefrontal cortex than those with *Neutral* *Cue*. FWE cluster-wise corrected, *p* < 0.05, cluster-defining threshold *p* = 0.001.
