# Supplementary material for: The expectations humans have of a pleasurable sensation asymmetrically shape neuronal responses and subjective experiences to hot sauce
Source: PLoS Biol. 2024 Oct 8;22(10):e3002818. doi: 10.1371/journal.pbio.3002818 (PMC11460714; doi:10.1371/journal.pbio.3002818)
Supplement: S3 Fig — (DOCX) [file pbio.3002818.s003.docx]

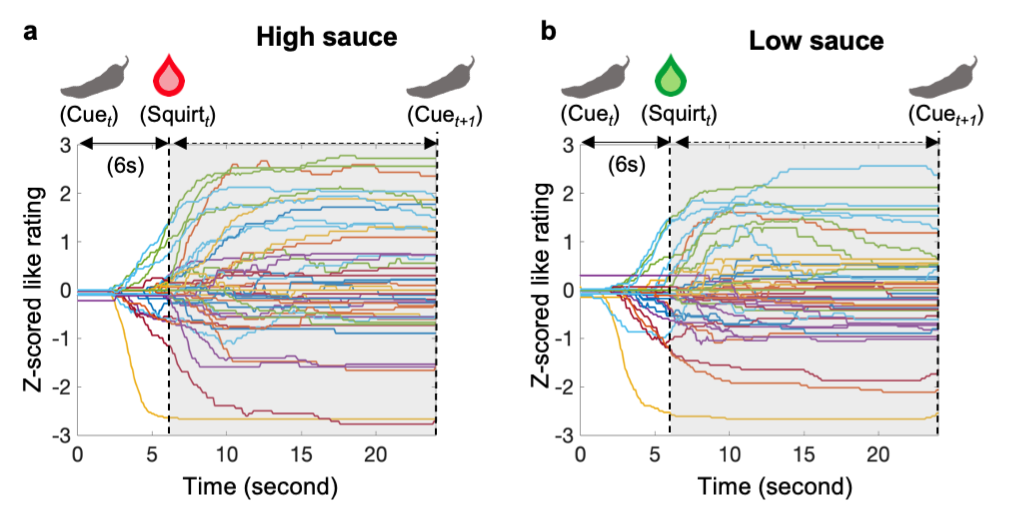


**S3 Fig**. Like ratings for each participant between heat and like ratings in Neutral Cue condition. **a**. The converted like rating for each participant for high-intensity hot sauce. **b**. The converted like rating for each participant for low-intensity hot sauce. Each line represents the response of a single participant.
