# Supplementary material for: The expectations humans have of a pleasurable sensation asymmetrically shape neuronal responses and subjective experiences to hot sauce
Source: PLoS Biol. 2024 Oct 8;22(10):e3002818. doi: 10.1371/journal.pbio.3002818 (PMC11460714; doi:10.1371/journal.pbio.3002818)
Supplement: S4 Fig — (DOCX) [file pbio.3002818.s004.docx]

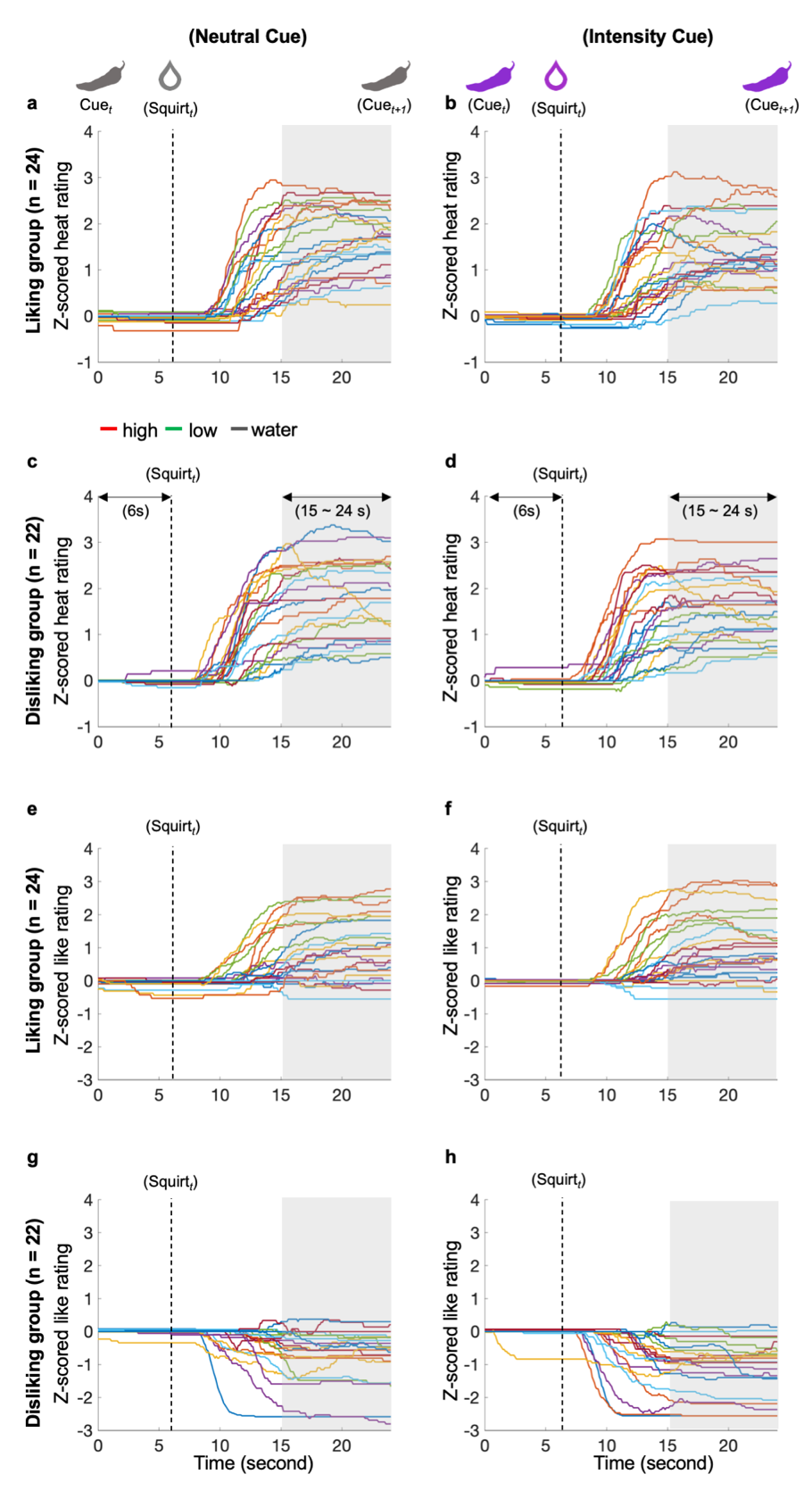


**S4 Fig.** **Individual behavioral rating trajectories. a** **& b.** The ratings of spiciness with *Neutral Cue* (a) and those with *Intensity Cue* (b) for each subject in the liking group. **c** **& d.** The ratings of spiciness with *Neutral Cue* (c) and those with *Intensity Cue* (d) for each subject in the disliking group. **e** **& f.** The ratings of preference with *Neutral Cue* (e) and those with *Intensity Cue* (f) for each subject in the liking group. **g** **& h.** The ratings of preference with *Neutral Cue* (g) and those with *Intensity Cue* (h) for each subject in the disliking group*.*
