# Supplementary material for: The expectations humans have of a pleasurable sensation asymmetrically shape neuronal responses and subjective experiences to hot sauce
Source: PLoS Biol. 2024 Oct 8;22(10):e3002818. doi: 10.1371/journal.pbio.3002818 (PMC11460714; doi:10.1371/journal.pbio.3002818)
Supplement: S5 Fig — (DOCX) [file pbio.3002818.s005.docx]

**
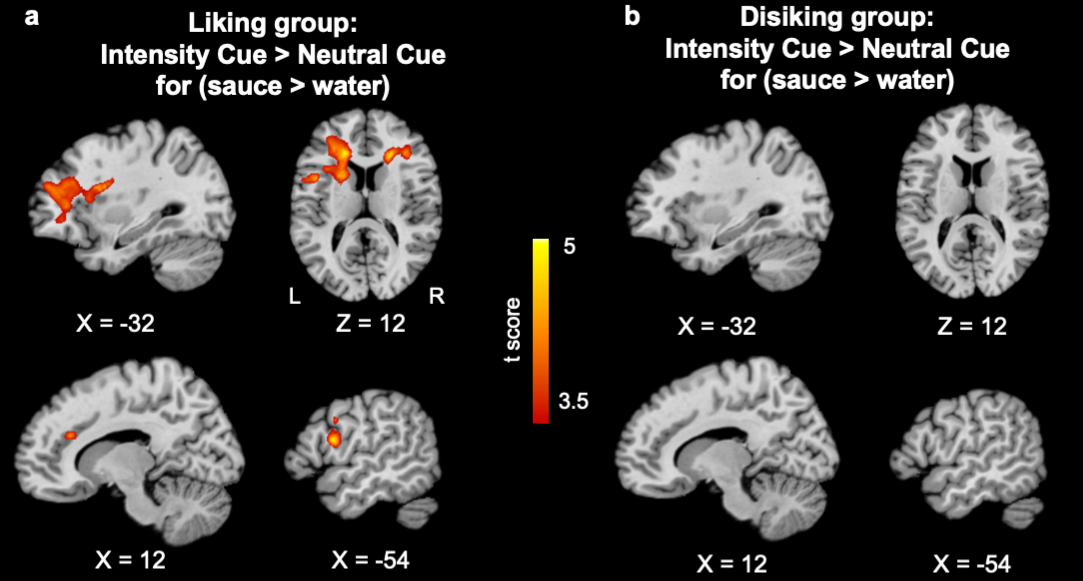
**

**S5 Fig**. Brain responses for the interaction of *Intensity Cue* (sauce > water) > *Neutral Cue* (sauce > water) at squirt delivery with saturated phase included in the first-level general linear model. **a.** Stronger brain responses for the liking group for sauce than water. **b.** For the contrast of sauce > water, the disliking group did not show different brain responses between *Intensity Cue* and *Neutral Cue* conditions. FWE cluster-wise corrected, *p* < 0.05, cluster-defining threshold *p* < 0.001.
