# Supplementary material for: The expectations humans have of a pleasurable sensation asymmetrically shape neuronal responses and subjective experiences to hot sauce
Source: PLoS Biol. 2024 Oct 8;22(10):e3002818. doi: 10.1371/journal.pbio.3002818 (PMC11460714; doi:10.1371/journal.pbio.3002818)
Supplement: S6 Fig — (DOCX) [file pbio.3002818.s006.docx]

**
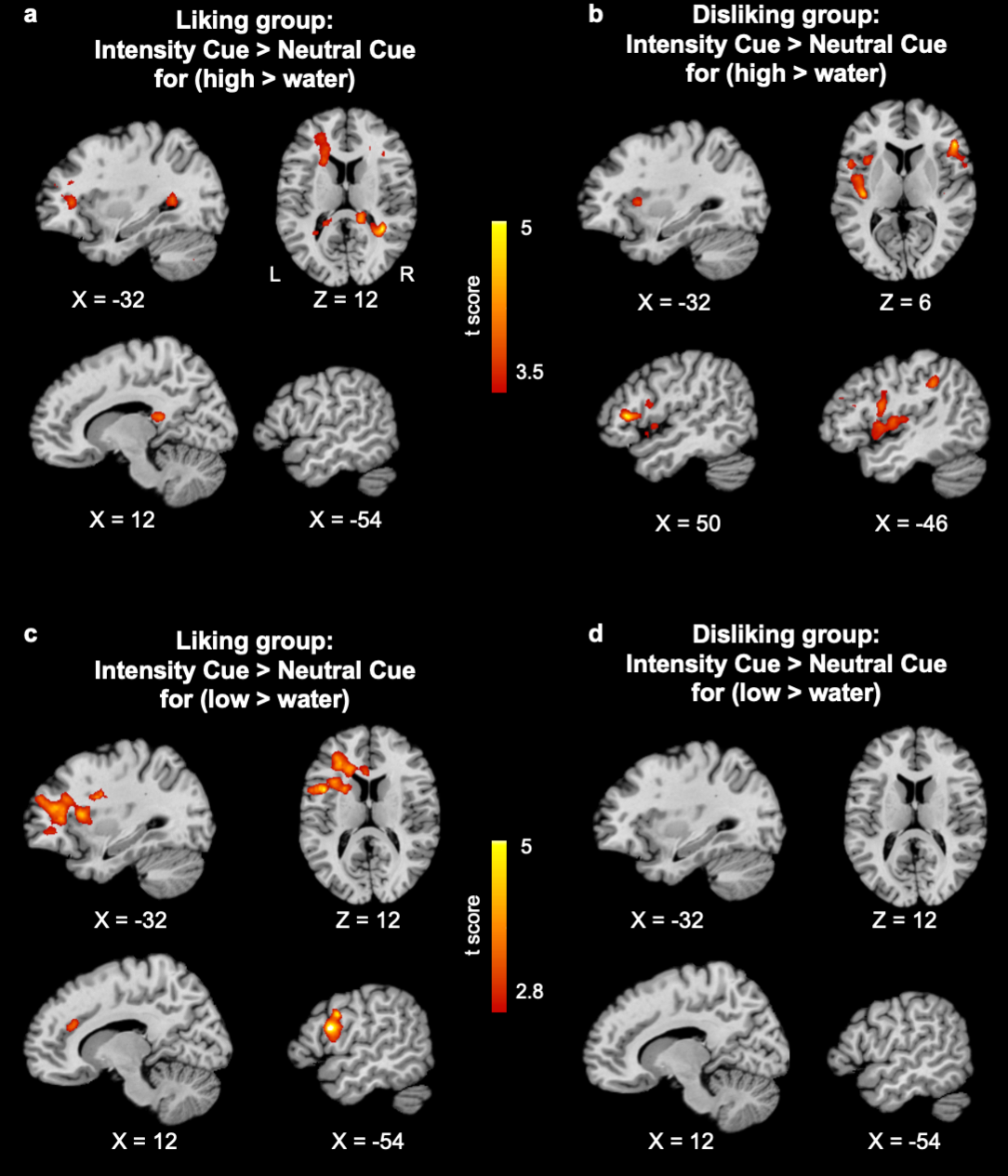
**

**S6 Fig**. Brain responses for the interaction of *Intensity Cue (sauce > water) > Neutral Cue (sauce > water)* at squirt delivery in each group, with high- and low-intensity hot sauces analyzed separately. **a & b.** Stronger brain responses in the liking group (a) and the disliking group (b) for high sauce versus water (*uncorrected p* < 0.005). **c & d.** Stronger brain responses in the liking group (c) and the disliking group (d) for low sauce versus water (FWE cluster-wise corrected, *p* < 0.05, cluster-defining threshold *p* < 0.001).
