# Supplementary material for: The expectations humans have of a pleasurable sensation asymmetrically shape neuronal responses and subjective experiences to hot sauce
Source: PLoS Biol. 2024 Oct 8;22(10):e3002818. doi: 10.1371/journal.pbio.3002818 (PMC11460714; doi:10.1371/journal.pbio.3002818)
Supplement: S7 Fig — Individual data for b and c are deposited in https://osf.io/cvjtd/?view_only=82aa9d97102c425f963ab1b4e52e8580. (DOCX) [file pbio.3002818.s007.docx]

**
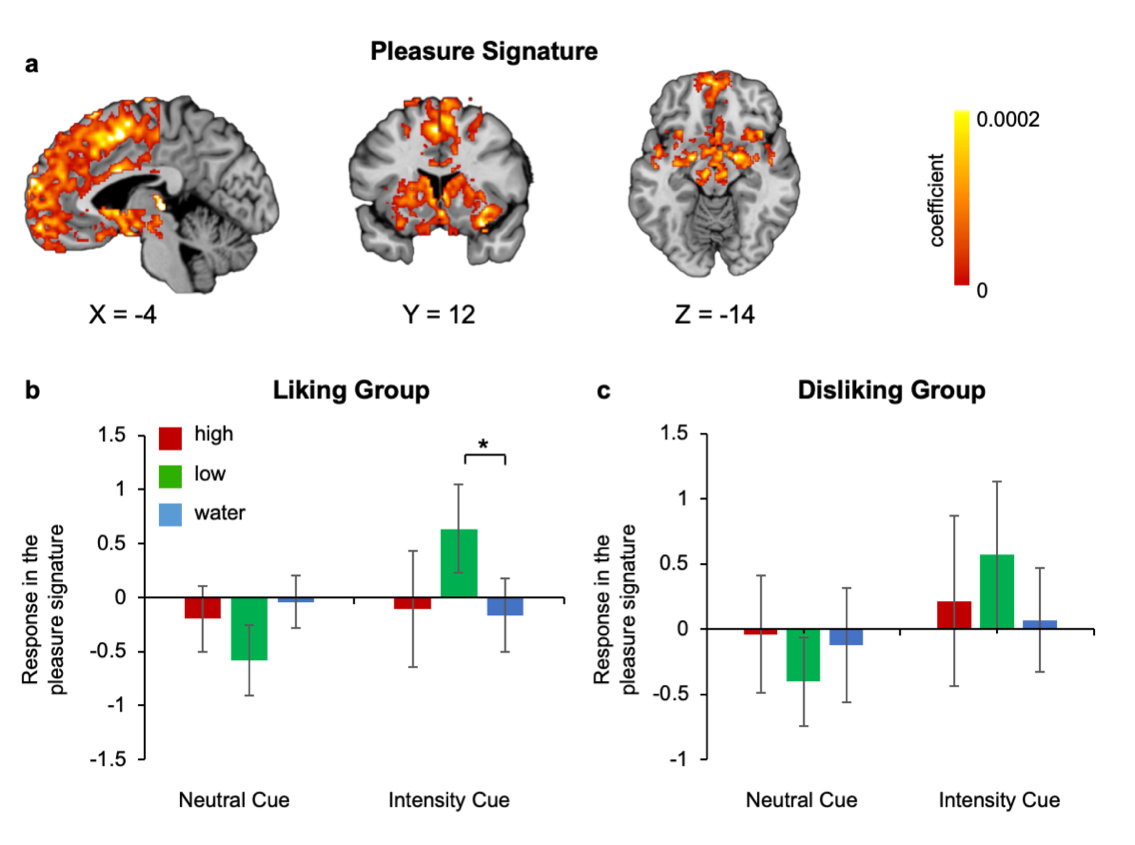
**

**S7 Fig**. Responses in the pleasant signature in participants grouped by the intrinsic preference for the spicy taste. **a**. The pleasant pain signature (Kragel et al., 2023; only voxels with positive coefficients) applied to each participant’s first-level general linear model to extract the average beta value in this mask. **b**. The responses to squirts in the pleasant signature for the liking group. Only with *Intensity Cue*, this group had stronger activations for low-intensity hot sauce than water. * *Uncorrected* *p* < 0.05.**c**. The responses to squirts in the pleasant signature for the disliking group. Individual data for **b** & **c** are deposited in https://osf.io/cvjtd/?view_only=82aa9d97102c425f963ab1b4e52e8580.
