# Supplementary material for: The expectations humans have of a pleasurable sensation asymmetrically shape neuronal responses and subjective experiences to hot sauce
Source: PLoS Biol. 2024 Oct 8;22(10):e3002818. doi: 10.1371/journal.pbio.3002818 (PMC11460714; doi:10.1371/journal.pbio.3002818)
Supplement: S8 Fig — (DOCX) [file pbio.3002818.s008.docx]

**
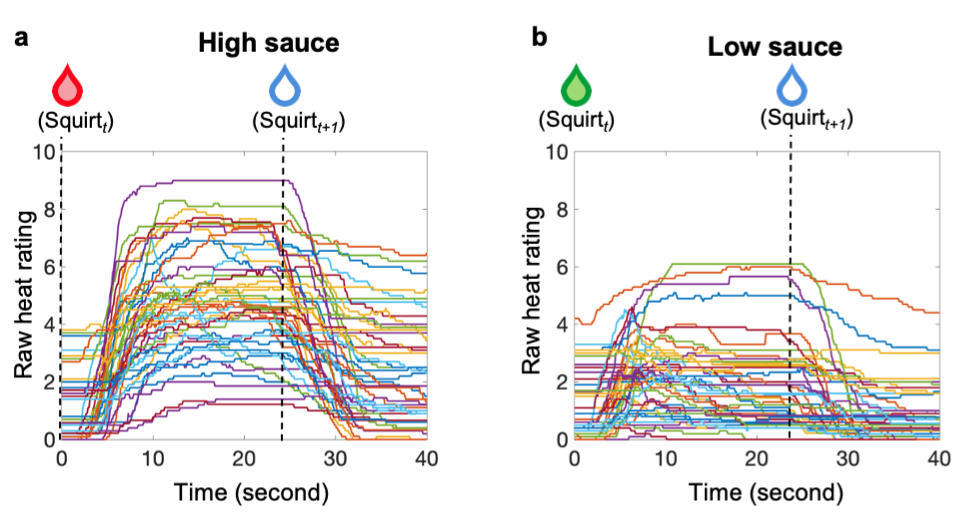
**

**S8 Fig**. Raw heat ratings for each participant. **a**. The raw heat rating for each participant for high-intensity hot sauce. **b**. The raw heat rating for each participant for low-intensity hot sauce. Each line represents the response of a single participant. Squirt*_t+1_* is always a rinse squirt.
