# Supplementary material for: The expectations humans have of a pleasurable sensation asymmetrically shape neuronal responses and subjective experiences to hot sauce
Source: PLoS Biol. 2024 Oct 8;22(10):e3002818. doi: 10.1371/journal.pbio.3002818 (PMC11460714; doi:10.1371/journal.pbio.3002818)
Supplement: S1 Table — (DOCX) [file pbio.3002818.s009.docx]

**S1 Table. Correlation coefficient and significance of heat and liking ratings in *Neutral Cue* condition for each participant**

| Group | Subject ID | *r* | *p* |
| --- | --- | --- | --- |
| Liking group | 1 | 0.05 | < .001 |
|  | 2 | 0.83 | < .001 |
|  | 4 | 0.79 | < .001 |
|  | 5 | 0.02 | 0.032 |
|  | 6 | 0.68 | < .001 |
|  | 8 | 0.50 | < .001 |
|  | 9 | 0.12 | < .001 |
|  | 10 | 0.15 | < .001 |
|  | 12 | 0.84 | < .001 |
|  | 13 | 0.28 | < .001 |
|  | 14 | 0.09 | < .001 |
|  | 19 | 0.87 | < .001 |
|  | 20 | 0.13 | < .001 |
|  | 21 | 0.05 | < .001 |
|  | 24 | 0.73 | < .001 |
|  | 26 | 0.75 | < .001 |
|  | 29 | 0.23 | < .001 |
|  | 31 | 0.58 | < .001 |
|  | 34 | 0.36 | < .001 |
|  | 38 | 0.18 | < .001 |
|  | 40 | 0.43 | < .001 |
|  | 41 | 0.47 | < .001 |
|  | 45 | 0.83 | < .001 |
|  | 47 | 0.76 | < .001 |
| Disliking group | 3 | -0.79 | < .001 |
|  | 7 | -0.47 | < .001 |
|  | 11 | -0.29 | < .001 |
|  | 15 | -0.41 | < .001 |
|  | 17 | -0.60 | < .001 |
|  | 18 | -0.16 | < .001 |
|  | 22 | -0.02 | 0.006 |
|  | 23 | -0.03 | < .001 |
|  | 25 | -0.54 | < .001 |
|  | 27 | -0.43 | < .001 |
|  | 28 | -0.84 | < .001 |
|  | 30 | -0.35 | < .001 |
|  | 32 | -0.63 | < .001 |
|  | 33 | -0.04 | < .001 |
|  | 35 | -0.06 | < .001 |
|  | 36 | -0.48 | < .001 |
|  | 37 | -0.17 | < .001 |
|  | 39 | -0.73 | < .001 |
|  | 42 | -0.40 | < .001 |
|  | 43 | -0.59 | < .001 |
|  | 44 | -0.18 | < .001 |
|  | 46 | -0.56 | < .001 |
