# Supplementary material for: The expectations humans have of a pleasurable sensation asymmetrically shape neuronal responses and subjective experiences to hot sauce
Source: PLoS Biol. 2024 Oct 8;22(10):e3002818. doi: 10.1371/journal.pbio.3002818 (PMC11460714; doi:10.1371/journal.pbio.3002818)
Supplement: S2 Table — (DOCX) [file pbio.3002818.s010.docx]

**S2 Table. Clusters with stronger activations for sauce > water with *Intensity Cues* than *Neutral Cues***

| **Brain Regions** |  | **MNI Coordinates** | | |  | **peak *t* value** | **Cluster Size** |
| --- | --- | --- | --- | --- | --- | --- | --- |
|  |  | **(mm)** | | |  |  |  |
|  |  | **x** | **y** | **z** |  |  | **(voxels)** |
| ***All subjects*** | | | | | | | |
| L AI, DLPFC | | -50 | 6 | 14 |  | 6.23 | 2526 |
|  |  | -30 | 38 | 16 |  | 4.96 |  |
|  |  | -34 | 12 | 8 |  | 4.92 |  |
| R AI, DLPFC |  | 54 | 8 | 10 |  | 5.56 | 1304 |
|  |  | 34 | 18 | 2 |  | 4.38 |  |
|  |  | 38 | -2 | 6 |  | 4.18 |  |
| R DLPFC |  | 38 | 32 | 18 |  | 4.10 | 752 |
|  |  | 42 | 36 | 24 |  | 4.02 |  |
|  |  | 44 | 44 | 14 |  | 3.97 |  |
| ***Liking Group*** | | | | | | | |
| L AI, DLPFC | | -22 | 26 | 4 |  | 5.27 | 2264 |
|  |  | -22 | 10 | 16 |  | 5.09 |  |
|  |  | -56 | 8 | 16 |  | 5.06 |  |
| R AI, dACC |  | 12 | 30 | 22 |  | 4.72 | 430 |
|  |  | 24 | 30 | 14 |  | 4.67 |  |
|  |  | 24 | 38 | 14 |  | 4.56 |  |
| ***Disliking Group*** | | | | | | | |
| None |  |  |  |  |  |  |  |

L, left; R, right; AI, anterior insula; DLPFC, dorsolateral prefrontal cortex, dACC, dorsal anterior cingulate cortex.
