# Supplementary material for: The expectations humans have of a pleasurable sensation asymmetrically shape neuronal responses and subjective experiences to hot sauce
Source: PLoS Biol. 2024 Oct 8;22(10):e3002818. doi: 10.1371/journal.pbio.3002818 (PMC11460714; doi:10.1371/journal.pbio.3002818)
Supplement: S3 Table — (DOCX) [file pbio.3002818.s011.docx]

**S3 Table. Reaction time in each condition**

| **Rating** | **Condition** | ***mean ± s. d.* (seconds)** | | ***p*** |
| --- | --- | --- | --- | --- |
|  |  | ***Neutral Cue*** | ***Intensity Cue*** |  |
| Heat rating | high sauce | 6.4 ± 2.4 | 6.1 ± 3.3 | 0.083 |
|  | low sauce | 9.2 ± 6.8 | 9.6 ± 14.5 | 0.827 |
|  | water | 6.5 ± 4.8 | 7.1 ± 6.7 | 0.479 |
| Like rating | high sauce | 8.8 ± 4.1 | 7.9 ± 3.7 | 0.028 |
|  | low sauce | 8.1 ± 3.7 | 7.5 ± 4.5 | 0.226 |
|  | water | 7.6 ± 3.9 | 6.6 ± 3.7 | 0.052 |

Note: reaction time was defined as the time from squirt delivery and the first button press after it. The *p* value indicates the significance of paired *t* tests.
