# Supplementary material for: The expectations humans have of a pleasurable sensation asymmetrically shape neuronal responses and subjective experiences to hot sauce
Source: PLoS Biol. 2024 Oct 8;22(10):e3002818. doi: 10.1371/journal.pbio.3002818 (PMC11460714; doi:10.1371/journal.pbio.3002818)
Supplement: S1 Text — (DOCX) [file pbio.3002818.s012.docx]

**S1 Text. Supplementary Methods**

**Instructions for participants**

Prior to the experiment, participants were given detailed instructions on how to use these scales. Participants were instructed as following:

*“The goal of today’s experiment is to understand how the human brain responds when people experience, or taste, different flavors.*

*In today’s experiment, you will taste different sauces while being scanned.*

*These sauces are store bought sauces.*

*For the purposes of the experiment, we cannot tell you what they are.*

*We can tell you that the company that produces these sauces is not sponsoring the experiment and is not going to directly gain or lose anything based on your data.*

*It is possible that you will taste sauces that may seem “spicy” or “hot” to you.*

*It is also possible that you will taste sauces that* ***do*** ***not*** *seem “spicy” or “hot” to you.*

*We do not know which of these sauces you will receive.*

*If this is a problem, you may choose to not participate.*

*While you taste these sauces, you will rate how the flavor tastes to you.*

*On the screen, you will see two scales, which you will adjust, using button presses with your left and right hand.*

*In a moment, we will show you the button boxes and explain how to use them.*

*For now, look at the screen.*

*You will use these two scales to answer the following two questions:*

*1) “How ‘spicy’ or ‘hot’ does your mouth feel?”*

*And…*

*2) “How much do you like the flavor that you taste?”*

*You will continuously answer these questions by adjusting the red bar to the appropriate level based on how you think the flavor in your mouth tastes.*

*We would like you to adjust these bars as the experience in your mouth changes: in other words not just when you receive a squirt and swallow, but throughout the entire task: while you receive a squirt, while you taste the sauce, during and after you have swallowed, and while you wait for the next squirt.*

*As you can see the top of the bars indicate the highest level of “spiciness” or “liking” and the bottom of the bars indicate the lowest level of “spiciness” or “liking”.*

*The bars can be adjusted across eleven levels ranging from zero to ten (zero is the lowest and ten is the highest).*

*You will use two button boxes, one in each hand, to adjust the levels of these red bars as the experience in your mouth changes.*

*If your mouth feels “hotter”, you will increase this bar.*

*If the “spiciness” goes away, you will lower this red bar in the appropriate direction.*

*At the same time,*

*If you “like” the flavor you taste you will increase this bar.*

*If you begin to dislike the flavor you taste then you will decrease this bar.*

*Throughout the experiment, you can raise or lower these bars to whatever level you feel best describe your level of experience.*

*During the scan, the bars may appear as you see here or on the opposite sides of the screen.*

*Once the experiment begins, the bars will not change location.*

*We will instruct you about which button controls which bar in a moment.*

*When the sauce is delivered to your mouth simply taste it and swallow the liquid.*

*You will not be given too much at one time, only enough to taste the flavor.*

*You will also not be told when the next squirt is coming, so be ready to receive the sauces at all times once the experiment begins.”*

**Salsa sauce lab test procedures**

**Extraction.** Salsa sauce samples were blended for 10 minutes to create a homogenous sample. Five grams of each sample were added to 25 mL of methanol in a 50 mL conical tube and homogenized for 3 minutes using a Fisherbrand PowerGen 1000 homogenizer. Samples were then centrifuged for 10 minutes at 15,000 g. For hot samples (high-intensity hot sauce), the supernatant was removed and diluted 1:10 in laboratory grade water. For mild samples (low-intensity hot sauce), the supernatant was removed and diluted 1:2 in laboratory grade water.

**ELISA Procedure.** Capsaicin concentrations were measured using a Beacon Capsaicin Plate kit (Beacon Analytical Systems, ME). All kit reagents and samples were warmed to room temperature. 100 uL of sample or standards (0.0, 0.1, 0.5, 2.0 ppm) were mixed with 100 uL of enzyme conjugate in their designated mixing wells. 100 uL of that mixture was then placed in antibody-coated wells, covered with parafilm, and incubated on a rotator for 10 minutes. Following incubation, each well was washed five times with distilled water. Following the final wash, 100uL of substrate was added and allowed to incubate for 10 minutes. 100uL of 1N HCl stop solution was then added to each well in the same order as the substrate. The plate was read using a Molecular Devices SpectraMax 384 Plus Microplate Reader at 450 nm.

**Concentration Calculation.** Samples and standards were processed and analyzed in duplicate. The average of the two was used to calculate the %B_0_. %B_0_ for each well was calculated using the following equation:

%B_0_ = (average OD of control, standard, or sample x 100) / (average OD of negative control)

The %B_0_ of each standard was graphed in GraphPad Prism using a semi-log graph. A best fit line was calculated and used to determine the capsaicin concentration for each sample.

**Behavioral Data Analysis Pooling All Participants Together**

To investigate the effect of expectation for spiciness, we compared the heat ratings for different types of squirts after *Neutral Cues* (run 1) and those after *Intensity Cues* (run 2). A repeated two-way analysis of variance (ANOVA) test of stimulus (within-subjects: high, low, water) × expectation manipulation (within-subjects: *Neutral Cue*, *Intensity Cue*) was done on the average heat ratings from 0 to 18 seconds after squirt delivery (i.e., 6 to 24 seconds after cue display). *Post hoc* tests were done to confirm the effect of expectation on each type of squirt.

A further inspection of the difference in the heat ratings between the *Intensity Cue* run versus the *Neutral Cue* run reveals two phases of the modulation by expectation (**S1c Fig**): a rising phase (7 to 11 seconds after cue display) and a saturated phase (15 to 24 seconds after cue display). We conducted another two ANOVA tests of sauce × expectation manipulation on the average heat ratings during the saturated phase.

Like rating was analyzed using the same approach.

**fMRI Data Analyses Pooling All Participants Together**

Pooling all participants together, we examined the possible interaction between stimulus and expectation manipulation by conducting a whole-brain contrast: (sauce___*_Intensity Cue_ – water__ Intensity Cue_) – (sauce*___*_Neutral Cue_* vs. water___*_Neutral Cue_*).

**fMRI Data Analyses taking account of the saturated phase**

To take into account the saturated phase as indicated by participants' ratings, we ran an additional analysis on the fMRI data. Specifically, we built first-level GLMs which include all visually and orally delivered stimuli, as well as motor responses, as stick regressors. Additionally, we included a regressor with onset at 15 seconds after cue display when the saturated phase began, with a duration of 9 s (from the start to the end of the saturated phase). Beta maps for the squirt delivery and the saturated phase were estimated respectively at each run for each participant and then entered into group-level analyses. After dividing participants into the liking group and the disliking group, we conducted a whole-brain contrast: *(sauce*___*_Intensity Cue_ – water__ Intensity Cue_) – (sauce*___*_Neutral Cue_* vs. water___*_Neutral Cue_*) within each group for both the squirt delivery event and the saturated phase.
