# Supplementary material for: The expectations humans have of a pleasurable sensation asymmetrically shape neuronal responses and subjective experiences to hot sauce
Source: PLoS Biol. 2024 Oct 8;22(10):e3002818. doi: 10.1371/journal.pbio.3002818 (PMC11460714; doi:10.1371/journal.pbio.3002818)
Supplement: S2 Text — (DOCX) [file pbio.3002818.s013.docx]

**S2 Text. Supplementary Results**

***Reported spiciness was modulated by expectations about spiciness pooling all participants together***

By comparing the average rating of experienced spiciness after *Neutral Cues* (**S1a Fig**) and those after *Intensity Cues* (**S1b Fig**) with all participants pooled together, we found a significant stimulus (high, low, water) × expectation manipulation (*Neutral Cue*, *Intensity Cue*) interaction, *F*(2,76) = 4.927, *p* = 0.014, $\eta_{p}^{2}$ = 0.097.

A further inspection of the difference in the heat ratings between the *Intensity Cue* run vs. *Neutral Cue* run reveals two phases of the modulation by expectation (**S1c Fig**): a rising phase (7 to 11 seconds after cue display) and a saturated phase (15 to 24 seconds after cue displayed). During the saturated phase, the stimulus × expectation manipulation was significant, *F*(2,79) = 7.438, *p* = 0.002, $\eta_{p}^{2}$ = 0.139. High (mean ± standard deviation; 1.45 ± 0.71) and low (0.20 ± 0.60) sauces with *Intensity Cues* were rated as less spicy than those with *Neutral Cues* (high: 1.64 ± 0.78, *p* = 0.049; low: 0.39 ± 0.66, *p* = 0.001), while water with *Intensity Cue* (-0.17 ± 0.20) was rated as significantly closer to baseline level than that with *Neutral Cue* (-0.29 ± 0.24), *p* = 0.007 (**S1d Fig**). This finding suggests that the expectation about spiciness modulated the relevant experience.

***Neural responses to high-intensity hot sauces were enhanced by the expectation of spiciness pooling all participants together***

We compared brain responses to sauces with *Intensity Cues* and those with *Neutral Cues* at the time of squirt delivery, with water tasting as a baseline. We found that the contrast of sauce (high and low sauces pooled) - water had stronger brain activations in the bilateral anterior insula (AI) and DLPFC when *Intensity Cues* were presented than that with *Neutral Cues* (**S2 Fig and S2 Table**).
